# *Pandoraea pnomenusa* Isolated from an Australian Patient with Cystic Fibrosis

**DOI:** 10.3389/fmicb.2016.00692

**Published:** 2016-05-11

**Authors:** Mark Ambrose, Roslyn C. Malley, Sanchia J. C. Warren, Sean A. Beggs, Oliver F. E. Swallow, Belinda McEwan, David Stock, Louise F. Roddam

**Affiliations:** ^1^School of Medicine, University of TasmaniaTasmania, TAS, Australia; ^2^Department of Pathology, Royal Hobart HospitalTasmania, TAS, Australia; ^3^Department of Microbiology and Infectious Diseases, Royal Hobart HospitalTasmania, TAS, Australia; ^4^Department of Paediatrics, Royal Hobart HospitalTasmania, TAS, Australia; ^5^Department of Respiratory and General Medicine, Royal Hobart HospitalTasmania, TAS, Australia

**Keywords:** *Pandoraea*, cystic fibrosis, infection, anaerobe, lung

## Abstract

*Pandoraea* species are considered as emerging pathogens in people with cystic fibrosis (CF). The contribution of these organisms to disease progression in CF patients is not fully understood owing in large measure to the scant reports in clinical and research literature describing their colonization of CF patients and their associated virulence determinants. In an effort to increase awareness and evidence for *Pandoraea* spp. infection in people with CF, and to stimulate research aimed at unraveling the pathogenic properties of *Pandoraea*, we report a case of a 26-year-old Australian (Tasmanian) man with CF who was chronically infected with *Pandoraea pnomenusa* for at least one year prior to his death from respiratory failure. In addition, we describe for the first time evidence suggesting that this bacterium is a facultative anaerobe and report on the availability of a whole genome sequence for this organism. To the best of our knowledge, this report represents only the second clinical case study of *P. pnomenusa* infection in the world, and the first in an Australian CF patient.

## Case report

In January 2013, a 26-year-old man with cystic fibrosis (CF, genotype homozygous F508del) and a history of chronic mucoid *Pseudomonas aeruginosa* lung infection was admitted to the Royal Hobart Hospital (RHH), Tasmania, Australia, with increasing malaise and left-sided pleuritic chest pain. The patient was at the time on his eighth day of out-patient intravenous antibiotic therapy for a respiratory exacerbation, which consisted of once daily tobramycin (560 mg), piperacillin/tazobactam (12 g of piperacillin component/24 h) as a continuous infusion, in addition to his long-term azithromycin (500 mg, orally three times a week) therapy.

**Figure 1:**
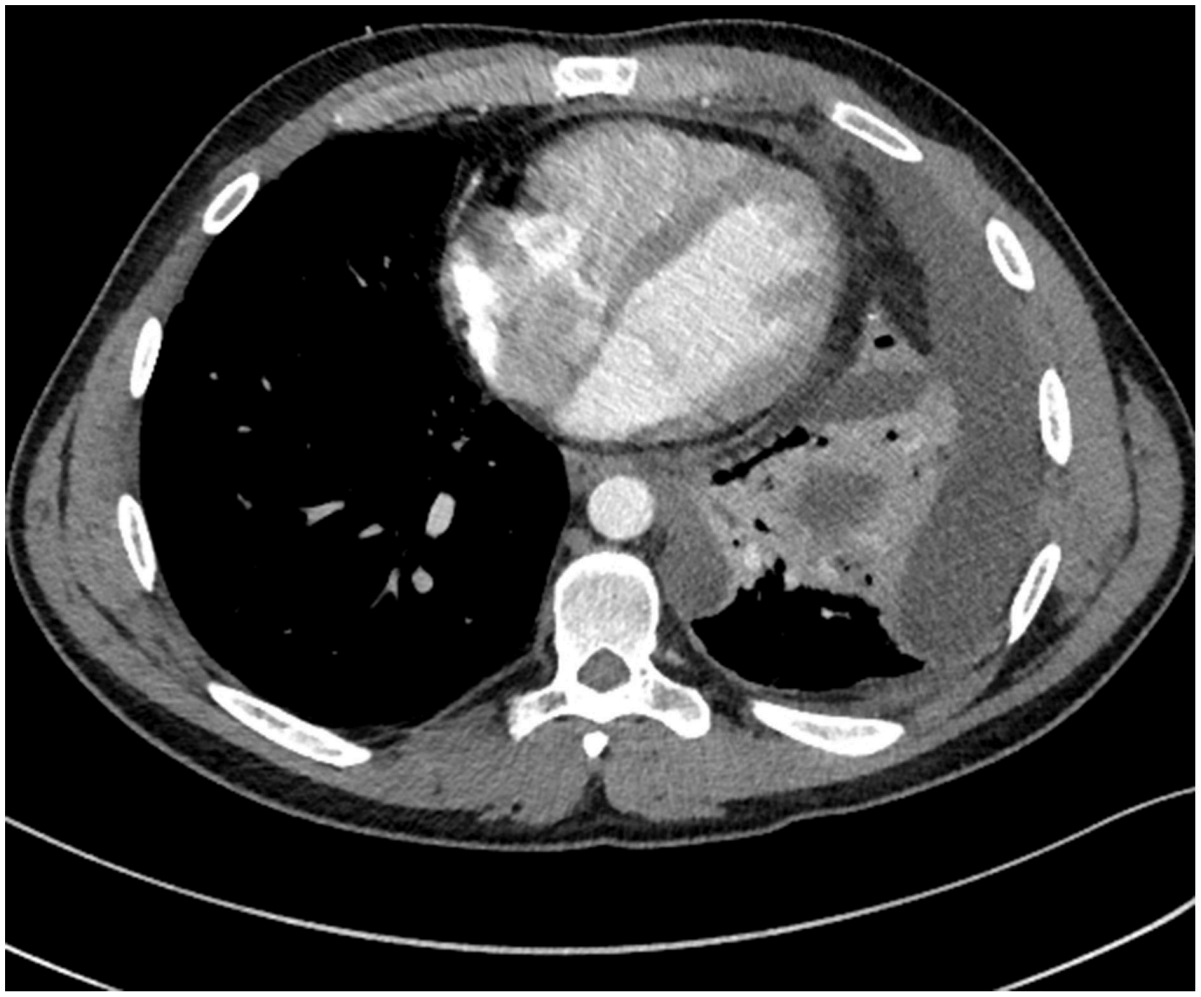
**A computed tomography (CT) scan showed a multi-loculated left pleural effusion**.

On the day of admission he was afebrile with an oxygen saturation of 97% on 4 L/min O_2_ via nasal cannulae, and his heart rate was 94 beats per minute with a blood pressure of 116/74 mm Hg. On physical examination, reduced breath sounds and inspiratory crackles were noted in the left lower zone. In addition, his white cell count was elevated (19.7 × 10^9^/L [normal range 3.5–11.0 × 10^9^/L]) with 80.2% neutrophils. A chest radiograph revealed a new and moderately large left pleural effusion. Computed tomography (CT) confirmed the multi-loculated left pleural effusion without contrast enhancement, as well as collapse/consolidation in the basal left upper and lower lobes (Figure 1). On the third day after admission a bronchoscopy and insertion of intercostal catheter (ICC) was performed in theater and serous fluid drained from the ICC. During the bronchoscopy copious purulent secretions were suctioned from the left bronchial tree and bronchial lavage performed. The bronchoscopy was complicated by respiratory failure, and consequently the patient was transferred to the intensive care unit (ICU) unit for mechanical ventilation. No bacteria or fungi were able to be recovered from the patient's pleural fluid samples collected at that time. In contrast, *P. aeruginosa* and *Candida albicans* were readily isolated from the bronchial pus. Moreover, *P. aeruginosa, C. albicans*, and what initially was determined to be a *Pandoraea apista* isolate, were all readily cultured from a sputum sample collected from the patient on the day of his admission to the RHH.

A repeat CT on the fourth day after the patient's admission showed a reduction in the volume of pleural fluid, but worryingly also the interval development of multiple bilateral foci of consolidation. His antibiotic regimen was altered to include imipenem (1 g IV, 6 hourly) and cotrimoxazole (1600/320 mg IV 6 hourly), extending the spectrum of antibiotic cover to include *Pandoraea* species. Due to the general lack of antibiotic susceptibility data available for *Pandoraea* species, the hospital recorded antibiotic susceptibility patterns of the recovered *Pandoraea* isolate were interpreted using EUCAST antibiotic breakpoint values that had previously been determined for other more common CF pathogens (imipenem susceptibility breakpoint for *P. aeruginos*a and cotrimoxazole susceptibility breakpoint for *Stenotrophomonas maltophilia*, for example).

On day five, video-assisted thoracoscopic surgery (VATS) was performed; exploration of the left pleural space revealed extensive fibrinopurulent material; and loculations were carefully divided and a decortication performed. Histology from the visceral pleural rind showed features of an organizing empyema. Despite the addition of empirical antimicrobials, namely vancomycin (1.5 g, 8 hourly) and caspofungin (70 mg loading dose, then 50 mg daily) there was no evident treatment response. Instead, the patient had persisting fevers and could not be weaned from ventilation.

A repeat bronchoscopy on day seven showed a much-reduced volume of large airways secretions. A contemporaneous CT showed progressive consolidation and increasing prominence of widespread ground-glass opacities, suspicious for the development of adult respiratory distress syndrome (ARDS). Bronchial washings also showed persistence of mucoid *P. aeruginosa, C. albicans*, and *Pandoraea*. In keeping with an advanced directive, active treatment was withdrawn and palliative care commenced. On the 11^th^ day after his admission to the RHH, the patient died.

## Microbiology

On a previous (December 2011) presentation to the RHH, a multiple antibiotic resistant isolate was recovered from the patient's sputum sample that was streaked out on a *Burkholderia cepacia*-selective medium. The isolate was presumptively identified as a non-mucoid *P. aeruginosa* but was not stored. In February 2012, the patient presented to a local private hospital and a similar multi-resistant sputum isolate was recovered. This isolate was retrospectively identified as *P. apista* by matrix-assisted laser desorption ionization-time of flight mass spectrometry (MALDI-TOF MS, Bruker Daltonics GmbH, Leipzig, Germany) with a score of 2.049, which was indicative of a reliable identification. The isolate was also submitted to the Microbiological Diagnostic Unit Public Health Laboratory (University of Melbourne, Australia) but 16S rRNA gene sequencing and phenotypic testing collectively were only able to identify the isolate as belonging to the genus *Pandoraea*.

In January 2013, the patient's forced expiratory volume in 1 second (FEV1) was lower (55% predicted) than his previous measurement (of 60%, late 2012). Following this diagnosis, the out-patient treatment regimen with tobramycin, piperacillin/tazobactam, and azithromycin was continued. A sputum culture obtained from the patient eight days prior to his presentation and admission to the RHH (in January 2013) showed that he had a persistent poly-microbial airway infection, including *P. aeruginosa, Staphylococcus aureus, Streptococcus milleri group, C. albicans*, and *P. apista*; the identity of this *P. apista* isolate was confirmed by the MALDI-TOF MS, at the RHH. The *P. apista* isolate was subsequently demonstrated to be susceptible to the antibiotics imipenem (by RHH staff) and cotrimoxazole but resistant to ceftazidime, ciprofloxacin, gentamicin, tobramycin, piperacillin-tazobactam, ticarcillin/clavulanic acid (timentin), aztreonam, ceftriaxone, meropenem, colistin, and trimethoprim (Table 1). It is noteworthy that the antibiotic susceptibility profile of this *P*. *apista* isolate was similar to that obtained for the initial multi-drug resistant isolate recovered from the patient's sputum sample (December 2011).

**Table 1:** ***P. pnomenusa* antibiotic susceptibility testing by disk diffusion assay**.

**Antibiotic**	**Disc content (μg)**	**Zone diameter breakpoint for Resistance (mm)**	**Inhibition zone diameter (mm)**	**Resistant (R)/Susceptible (S)**
Meropenem	10	^a^18	0	R
Amikacin	30	^a^15	0	R
Ciprofloxacin	5	^a^22	0	R
Ceftazidime	10	^a^16	0	R
Ticarcillin-clavulanic acid	85	^a^18	8	R
Gentamicin	10	^a^15	0	R
Trimethoprim	5	–	0	–
Aztreonam	30	^a^16	0	R
Tobramycin	10	^a^16	0	R
Cefepime	30	^a^18	0	R
Piperacillin-tazobactam	36	^a^18	16	R
Trimethroprim-Sulphamethoxazole	25	^b^16	28.5	^c^S

a Pseudomonas spp. breakpoint.

b Stenotrophomonas maltophilia breakpoint.

c Minimum inhibitory concentration (MIC) of trimethoprim-sulfamethoxazole (cotrimoxazole, 12.5 μg/ml) determined by the broth dilution method.

Interestingly, the CF lung environment is characterized by the presence of hypoxic micro-niches yet the ability of *Pandoraea* to colonize these niches is unknown. We demonstrated that the *P. apista* isolates were able to grow under anaerobic conditions. BHI agar plates supplemented with 1% potassium nitrate were inoculated with *Pandoraea* cultures and incubated in an anaerobic jar (containing an anaerobic sachet [AN0035; AnaeroGen, Oxoid, UK] and anaerobic indicator) at 37°C for five days. While growth of the *Pandoraea* cultures was scant under these conditions, it was nonetheless reproducible. Similarly, Daneshvar et al. (2001) also reported on the growth of nine *Pandoraea* isolates in a candle jar atmosphere. Collectively, these data would seem to indicate that at least some *Pandoraea* species might well be facultative anaerobes.

To gain an insight into the virulence mechanisms of *Pandoraea* species, it was decided to sequence the genomes of the two *P*. *apista* isolates recovered from this patient 11 months apart. Much to our surprise, the 16S rDNA sequence component of the genomes from both the February 2012 and January 2013 isolates identified them as *Pandoraea pnomenusa* (Ee et al., 2015). It is necessary to point out here that misidentification of *Pandoraea* species by MALDI-TOF MS alone is not uncommon because this method relies upon databases that are generated for microorganisms routinely recovered from clinical specimens. However, *Pandoraea* species are not routinely isolated or identified by the conventional microbiological methods available in many diagnostic laboratories; in turn, this makes creation of databases for these organisms that are necessary for accurate MALDI-TOF MS analyses somewhat problematic (Fernández-Olmos et al., 2012a). On review of the original MALDI-TOF MS identification scores, *P. apista* scored highest (2.090) followed by *P. pulmonicola* (1.817) and *P. pnomenusa* (1.614). The low score generated for *P. pnomenusa* (i.e., < 1.7) would likely have been interpreted as being unreliable for species determination. The results described in this case report further underscore the limitations of using MALDI-TOF MS for differentiating *Pandoraea* species and suggest that until such time comprehensive databases are available, accurate identification of *Pandoraea* species will be dependent upon molecular characterization (Coenye and LiPuma, 2002; Schneider and Bauernfeind, 2015).

## Discussion

Cystic fibrosis (CF) is an autosomal recessive disease, which can be caused by any one of the over 2000 mutations known to affect the cystic fibrosis transmembrane conductance regulator (CFTR) gene located on human chromosome 7. These mutations lead to an absent or dysfunctional chloride ion channel in respiratory epithelial cells, which in turn results in the accumulation of viscous respiratory secretions in the lungs of people with CF (Wilschanski et al., 1995). As a consequence, the airway of CF patients is intrinsically susceptible to colonization by a plethora of respiratory pathogens, and from very early on in life is colonized by bacterial pathogens including *S. aureus* and *P. aeruginosa* (Renders et al., 2001). These infections inevitably lead to pulmonary exacerbations and lung function decline. Despite aggressive antibiotic treatment, infection by *P. aeruginosa* eventually becomes chronic and is responsible for much of the morbidity and most of the mortality of people with CF (Hauser et al., 2011). Intriguingly, many “new” microorganisms are being isolated from and identified in respiratory secretions collected from CF patients due in part to improved laboratory diagnostic methods, although their contributions to disease progression are not yet known.

One group of bacteria currently considered to be emerging CF pathogens belongs to the genus *Pandoraea*. The genus *Pandoraea* was described by Coenye et al. (2000) to differentiate them from other already well-known CF pathogens, including *Pseudomonas* and two closely related Gram-negative rods, *Burkholderia* and *Ralstonia* species. In fact, phenotypic methods used by many microbiology laboratories commonly lead to the misidentification of *Pandoraea* species as either *Burkholderia* or *Ralstonia* species (Henry et al., 2001; Fernández-Olmos et al., 2012b). Different species of *Pandoraea* have been described, including *P*. *apista, P*. *norimbergenesis, P*. *pnomenusa, P*. *pulmonicola*, and *P*. *sputorum*. There is great genotypic and phenotypic diversity within species of *Pandoraea* but most have thus far been demonstrated to be aerobic, non-spore forming, non-nitrate-reducing, non-lactose-fermenting, Gram-negative rods with a single polar flagellum (Stryjewski et al., 2003). In addition, *Pandoraea* have been isolated from environmental samples (soil, water; Coenye et al., 2000), as well as from wound sites and variety of other clinical specimens including sputum, lung tissue, and urine (Daneshvar et al., 2001; LiPuma, 2010). Importantly, *P. apista* has been isolated from the lungs and blood cultures of CF patients (Stryjewski et al., 2003; Johnson et al., 2004), demonstrating the invasive potential of this *Pandoraea* species. However, there are few reports in the medical literature describing the contribution of *Pandoraea* to disease progression in CF patients. On this line, Jørgensen et al. (2003) and Fernández-Olmos et al. (2012a) independently showed that chronic bronchopulmonary colonization by either *P*. *apista* or *P*. *sputorum* was associated with frequent exacerbations and lung function decline in CF patients. Furthermore, Caraher et al. (2008) demonstrated *in vitro* that infection of lung cells by *P*. *sputorum* and other *Pandoraea* species induced a strong proinflammatory response, with elevated interleukin-6 (IL-6) and interleukin-8 (IL-8) production. However, the precise virulence mechanism(s) of *Pandoraea* responsible for provoking proinflammatory responses in cells and leading to chronic infection and disease in CF, as well as non-CF patients, remain to be fully determined.

To gain an insight into the potential virulence mechanisms of *Pandoraea*, our research group recently sequenced the whole genome of the two *P. pnomenusa* (initially identified as *P. apista*) isolates recovered 11 months apart from the patient being discussed in this case report. Sequencing results showed that the genomes of the two isolates were very similar, and differed only by one gene that apparently encodes a predicted phage tail protein (Ee et al., 2015). When we compared the genome sequence of these clinical isolates to that of the environmental *P. pnomenusa* strain RB38, we found that the clinical isolates possessed 152 unique genes (most of which were virulence genes) and were missing 87 genes unique to the *P. pnomenusa* strain RB38. Many of the 130 genes (~105 open reading frames [ORFs]) predicted to be associated with virulence and defense are indicated to be involved with resistance to antibiotics and toxic compounds (Ee et al., 2015), which is perhaps not too surprising given that the *P. pnomenusa* isolates were demonstrated to be resistant to multiple antibiotics. Major advances in our understanding of the role(s) of *Pandoraea* in disease progression in CF rest upon knowing the virulence properties of these organisms. In this context, the whole genome sequences for *P. pnomenusa* generated by our research group (Ee et al., 2015) and others (Lim et al., 2016) provide a valuable resource, that could be easily mined by researchers interested in forming a better understanding of *Pandoraea* colonization in people with CF and discovering potential therapeutic targets. In addition, our results demonstrating that *P. pnomenusa* is a facultative anaerobe are significant. It is well established that hypoxic micro-niches are present in the CF lung, and that oxygen gradients across the thick airway mucus (Worlitzsch et al., 2002; Tunney et al., 2008) and oxygen consumption by polymorphonuclear cells and bacteria (Kolpen et al., 2010) potentiate microaerophilic and even anaerobic growth. It is therefore of high clinical significance that *P. pnomenusa* seems to be able to grow under anaerobic conditions, given that anaerobic growth appears to be associated with increased persistence and antibiotic resistance in other facultative anaerobes that infect the CF lung (Schobert and Tielen, 2010).

## Concluding remarks

This case report further highlights the limitations associated with using MALDI-TOF MS and phenotypic-based testing for the identification of uncommon pathogens, and serves as an important caution for laboratories relying on these two methodologies for accurate identification of species of *Pandoraea*. Without consistent and reliable species level identification of multidrug-resistant *Pandoraea*, the clinical significance of these organisms will remain difficult to determine.

## Author contributions

MA and LR substantially contributed to the conception of the work, the acquisition, analysis and interpretation of data and drafting and critically revising the work. MA and LR also have final approval of the version to be published and agree to be accountable for all aspects of the work. RM, SW, SB, OS, BM, DS substantially contributed to the acquisition, analysis and interpretation of data and in drafting and critically revising the work.

## Funding

This work was funded by a Royal Hobart Hospital Research Foundation (RHHRF) grant number R22664.

### Conflict of interest statement

The authors declare that the research was conducted in the absence of any commercial or financial relationships that could be construed as a potential conflict of interest.
